# Adaptive Evolution of *Staphylococcus aureus* during Chronic Endobronchial Infection of a Cystic Fibrosis Patient

**DOI:** 10.1371/journal.pone.0024301

**Published:** 2011-09-02

**Authors:** Paul R. McAdam, Anne Holmes, Kate E. Templeton, J. Ross Fitzgerald

**Affiliations:** 1 The Roslin Institute and Centre for Infectious Diseases, Royal (Dick) School of Veterinary Studies, University of Edinburgh, Easter Bush, Midlothian, United Kingdom; 2 Microbiology, Royal Infirmary of Edinburgh, Edinburgh, United Kingdom; Stanford University School of Medicine, United States of America

## Abstract

The molecular adaptation of *Staphylococcus aureus* to its host during chronic infection is not well understood. Comparative genome sequencing of 3 *S. aureus* isolates obtained sequentially over 26 months from the airways of a cystic fibrosis patient, revealed variation in phage content, and genetic polymorphisms in genes which influence antibiotic resistance, and global regulation of virulence. The majority of polymorphisms were isolate-specific suggesting the existence of an heterogeneous infecting population that evolved from a single infecting strain of *S. aureus.* The genetic variation identified correlated with differences in growth rate, hemolytic activity, and antibiotic sensitivity, implying a profound effect on the ecology of *S. aureus*. In particular, a high frequency of mutations in loci associated with the alternate transcription factor SigB, were observed. The identification of genes under diversifying selection during long-term infection may inform the design of novel therapeutics for the control of refractory chronic infections.

## Introduction

*Staphylococcus aureus* causes chronic, recurrent endobronchial infections of patients with cystic fibrosis (CF) during childhood [1], [2]. Impaired mucociliary clearance, insufficient aeration of the paranasal sinuses and nasal polyps, along with frequent hospital visits mean CF patients are at particularly high risk of *S. aureus* infections [3], [4]. In CF patients, it is the oropharynx rather than the anterior nares which is the predominant site of *S. aureus* infection and persistence [5] and the upper airways have been identified as a reservoir allowing for recurrent infection of the lower airways by the same clone [6]. Further, longitudinal studies have shown that isolates belonging to the same lineage are repeatedly recovered from most CF patients, indicating chronic infection with a single strain [3], [7]. During chronic infection, *S. aureus* is subjected to numerous selective pressures resulting from antibiotic interventions, the host immune system and co-infection of the airways with other microorganisms [8]. Adaptive strategies of *S. aureus* are thought to involve the emergence of antibiotic-resistant, low-virulence, persistent phenotypes [1], [9]. Of note, a switch to small colony variants (SCV) is often observed in persistent infections and is associated with higher levels of resistance to antibiotics [9], [10], [11], [12]. In addition, hyper-mutable *S. aureus* strains with a defective DNA mismatch repair system have been isolated from CF infections which promote adaptation to encountered selective pressures [13], [14], [15]. However, the molecular basis for the adaptation of *S. aureus* to the CF airways is not well understood and lags behind research into the CF pathogen *Pseudomonas aeruginosa* [16]. For example, a comparative genomic study revealed evidence for selection acting across the whole *P. aeruginosa* genome, reflected in an adapted virulence phenotype with loss of function of factors required for acute infection but enhanced ability to cause chronic inflammation [17]. Previous genome-scale analyses of *S. aureus* evolution during infection revealed that most mutations were the result of the strong selective pressure of antibacterial chemotherapy but may also be associated with promoting persistence [18], [19].

Here, we performed a comparative genomic analysis of sequential *S. aureus* isolates from a single CF patient to investigate *in vivo* adaptive diversification. The genetic and phenotypic variation identified among the isolates indicates an adapting infecting population which is genetically and phenotypically heterogenous. Overall, the study provides for the first time, a genome-wide, high resolution insight into the evolution of *S. aureus* during chronic infection of the cystic fibrosis airways.

## Methods

### Bacteria, growth conditions and phenotypic analysis

Sequential *S. aureus* isolates ED83, ED84 (11 months) and ED86 (26 months) were obtained from the sputum of a CF patient over a 26-month period and were previously characterized by pulse field gel electrophoresis (PFGE), capsule- and phage-typing to infer the clonal relatedness of the isolates [7]. *S. aureus* isolates were cultured in Tryptic Soy Broth (TSB), chemical defined media (CDM) and CDM lacking threonine as previously described [20]. Hemolytic activity was assessed after overnight culture of isolates at 37°C on tryptic soy agar (TSA) containing sheep blood. Sensitivity to antibiotics including Penicillin, Oxacillin, Gentamycin, Kanamycin, Tobramycin, Ciprofloxacin, Erythromycin, Clindamycin, Linezolid, Teicoplanin, Vancomycin, Tetracycline, Tigecycline, Nitrofurantoin, Fusidic acid, Mupiricin, Chloramphenical, Rifampicin, and Trimethoprim, was carried out using the Vitek 2 system (bioMérieux, Basingstoke, UK).

### Genome sequencing and analysis

Whole-genome sequencing was performed using the Illumina Genome Analyzer II platform (36 base-pair paired end (ED83) or single end reads (ED84 and ED86). High quality reads were aligned against the MRSA252 genome (accession number NC_002952) using Maq v0.7.1 (http://maq.sourceforge.net). A mean coverage of 34.2 was achieved (range 25.7–44.1). Point mutations and short indels were identified using Maq and Novoalign (http://www.novocraft.com/main/index.php), and confirmed by Sanger sequencing. Sequence reads unmapped to the reference sequence were assembled *de novo* using the program Velvet v0.7.63 (http://www.ebi.ac.uk/~zerbino/velvet), and protein-coding regions were identified through a BLASTx search against the NCBI non-redundant protein database. Comparative genomic analysis was performed using BRIG v0.71 (http://sourceforge. net/projects/brig) and IGV v1.5.06 (http://www.broadinstitute.org/software/igv) to identify differences in gene content between the isolates. Draft genomes have been deposited in the NCBI SRA database (accession number SRA038522.2).

### Phylogenetic and statistical analysis

Phylogenetic reconstruction was performed with RAxML v7.2.6 (http://wwwkaramer.in.tum.de/exelixix/software.html) for all sites in the core genome, using a GTR model of nucleotide substitution with the gamma model of rate heterogeneity. Support for nodes was assessed using 1000 bootstrap replicates. Loci accumulating a greater number of mutations per site in comparison to the rest of the genome were identified by a one-tailed Welch's T-test.

## Results and Discussion

**Figure 1:**
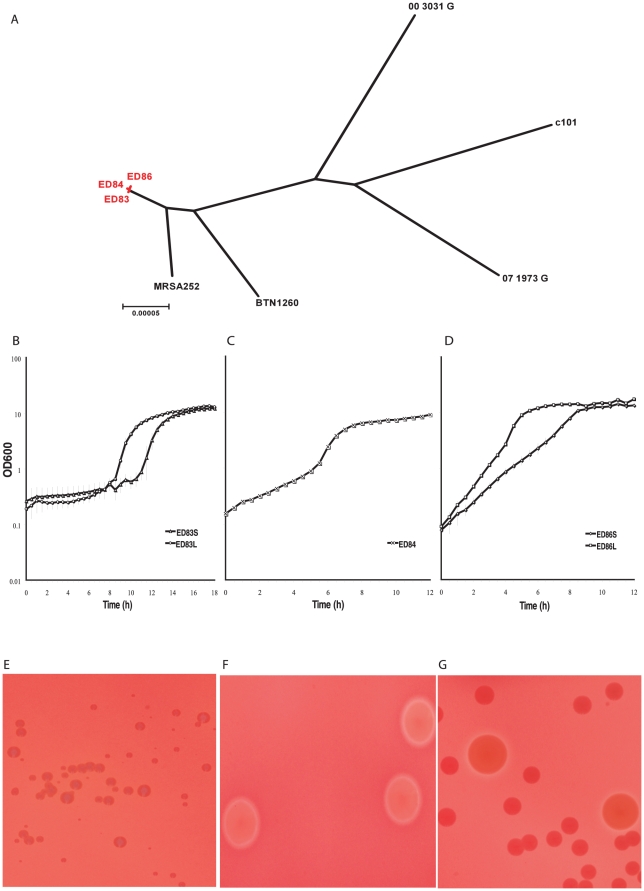
Genetic and phenotypic heterogeneity among sequential *S. aureus* CF isolates. (**A**) Maximum likelihood phylogeny based on core genome point mutations for the 3 CF isolates in comparison to 5 additional CC30 isolates. MRSA252 is representative of hospital associated epidemic ST36 strains [31], BTN1260 and c101 are methicillin sensitive (MSSA) ST30 isolates from a global collection [32], 00_3031_G and 07_1973_G are a community associated MSSA and a hospital associated MRSA from Scotland, respectively (unpublished data). Scale represents substitutions per site. Growth curves in threonine-depleted CDM for (**B**) smaller (S) and larger (L) colony variants of ED83, (**C**) ED84 and (**D**) smaller (S) and larger (L) colony variants of ED86. Hemolytic activity on sheep erythrocytes for **(E)** colony variants of ED83, **(F)** ED84 and **(G)** colony variants of ED86.

In a previous study, CF patients with persistent colonization of the airways by single *S. aureus* clones were identified by a combination of PFGE, capsule- and phage-typing of sequential isolates [7]. In order to examine the microevolution of *S. aureus* during chronic infection of the CF airways, the genomes of 3 of the sequential isolates obtained over 26 months from the airways of a persistently colonized CF patient were sequenced. Genome sequence analysis revealed each of the 3 isolates to be of multi-locus sequence type 30 (ST30), a globally successful epidemic clone, but a total of 23 point mutations and 15 indels were identified among the 3 isolates, including 31 intragenic and 6 intergenic polymorphisms (Table S1). Phylogenetic analysis of the 3 sequential CF isolates in comparison to 5 representative strains of the same clonal complex (CC30) based on core genome point mutations indicates that the 3 CF isolates form a very narrow, distinct clade within the CC30 tree, consistent with their evolution from a single infecting progenitor strain (Fig. 1). Of note, all 23 point mutations and 14 of the 15 indels identified among the 3 isolates were strain-specific indicating clonal diversification of the original infecting strain leading to a genetically heterogenous infecting population (Table S1). Of the 391 polymorphisms (376 point mutations and 15 indels) shared by the core genomes of all 3 isolates compared to the reference genome, none resulted in pseudogenes, and there were no mutations in the methyl-mismatch repair system (MutS and MutL) suggesting that the strains are not hypermutable. The great majority of point mutations (95.7%) were non-synonymous, consistent with previous studies that have reported a high ratio of non-synonymous to synonymous point mutations within clonal complexes, in contrast to a higher frequency of synonymous point mutations between clonal complexes [21]. These data support the hypothesis that mildly deleterious mutations are maintained in the short term. However, the high proportion of non-synonymous mutations are likely, at least in part, to be the result of strong selective pressures that exist in the unique environment of the CF airways. In particular, we discovered point mutations and indels in genes influencing global regulation, virulence, metabolism, and antibiotic resistance. For example, in the last sequential isolate (ED86), 2 amino acid-altering point mutations were identified in the *fusA* gene encoding elongation factor G (EF-G) including a H457Y amino acid replacement previously demonstrated to confer fusidic acid resistance [22]. Although the mutation is associated with a marked impairment in the biological fitness of *S. aureus*, the growth phenotype of ED86 compares favorably with that of the fusidic acid-susceptible isolates ED83 and ED84 in TSB, CDM and threonine-depleted CDM (Fig. 1), presumably due to a compensatory function of the second observed mutation, T187I, as previously reported for fusidic acid resistance strains of *S. aureus* [22]. Consistent with the presence of these mutations, isolate ED86 was resistant to fusidic acid. All 3 isolates were resistant to penicillin but were sensitive to each of the other antibiotics tested (data not shown).

Several of the identified mutations were in genes which may impact on virulence. For example, the branched chain amino acid binding pocket of the global regulator CodY [23] is invariant in all *S. aureus* genome sequences available in the public databases (data not shown). The gene encoding CodY in the third sequential isolate ED86 contains a non-synonymous point mutation resulting in a M65I amino acid replacement in the wall of the hydrophobic binding pocket which forms prominent hydrophobic interactions with the side chains of the branched chain amino acid ligand [24]. We speculate that the observed mutation may affect the function of CodY, which is a potent repressor of virulence [20], [23]. Of note, the ED86 isolate containing the M65I replacement has increased growth rate in minimal media deficient in threonine in comparison to isolates ED83 and ED84 which both contain wild type CodY alleles, a growth characteristic demonstrated previously for a CodY *S. aureus* mutant (Fig. 1)[20].

In addition, multiple, independent indel polymorphisms were observed in all 3 isolates which would influence expression of SigB, a regulator of the general stress response which influences the expression of virulence factors. Specifically, a frameshift mutation resulting in a *sigB* pseudogene in strain ED83, a frameshift mutation in SpoVG, a proposed modulator of SigB activity [25], and an 18 bp in frame deletion in the *rsbU* regulator of SigB in ED84, were identified. Modeling of the ED84 *rsbU* allele with the crystal structure of the *Bacillus subtilis* RsbU protein (PDB accession number 2J70) suggests that the 18 bp deletion is likely to impair the phosphatase activity of RsbU (data not shown). In addition, SpoVG and the histidine kinase ArlS, which have been shown to impact SigB-dependent capsule formation [25], are affected by a non-synonymous point mutation and a frameshift insertion respectively in the ED86 isolate. The parallel evolution of mutations at SigB-associated loci in the 3 isolates represent a 119-fold increase in mutation rate when compared to the mutation rate across the whole genome (p<0.05), suggesting that a strong selective pressure is acting on these loci leading to beneficial phenotypes adapted to the environment of the CF airways. Several previous studies have identified a role for SigB in the development of SCVs and in the regulation of transcription of virulence factors during infection of CF patients [9], [26], [27]. Our data suggest that there may be selective pressure for attenuation or loss of SigB expression during long-term infection. In order to further investigate this phenomenon, we determined the DNA sequence for SigB-associated loci *rsbU*, *rsbV*, *rsbW*, *sigB*, *spoVG*, *arlS*, and *arlR* among a panel of 16 *S. aureus* isolates from 4 CF patients persistently colonized with *S. aureus* [7]. However, only 2 mutations (non-synonymous substitutions in the *spoVG* and *arlR* loci in different isolates) were observed, and no loss of function mutations were identified at any of the loci examined (data not shown). Taken together, these data suggest that the elevated mutation rate in SigB-associated loci in *S. aureus* isolates ED83, ED84 and ED86 is related to the genetic background of the strain or to patient-specific selective pressures, and is not broadly applicable to all CF *S. aureus* isolates. Of note, clinical isolates of *S. aureus* with SigB-deficiency have been reported previously [28]. Previously, Entenza and colleagues investigated the role of SigB in a rat model of infective endocarditis and discovered increased bacterial densities of SigB-deficient strains after prolonged infective endocarditis infection suggestive of a selective pressure for reduced SigB activity during infection [29].

In order to examine the collective functional impact of the identified polymorphisms we examined hemolytic activity, and growth of each of the isolates in nutrient-replete and -deplete conditions. No difference in growth was observed between the 3 isolates in TSB (data not shown) but differences in growth rate were observed in CDM and CDM deficient in threonine (Fig. 1). Further, sub-culturing of single colonies of isolates ED83 and ED86 onto TSA containing sheep erythrocytes resulted in 2 distinct colony sizes which differed in hemolytic activity (large, hemolytic and small, non-hemolytic) for each isolate revealing an unstable *in vitro* phenotype (Fig. 1).

In addition to polymorphisms of the core genome, variation in the accessory genome of the isolates was observed. Examination of the genome sequence of isolate ED83 revealed 3 prophages including a 48.3 kb phage related to φSa2 of MRSA252, a 43.5 kb β-converting phage encoding the secreted virulence factors staphylococcus enterotoxin A (SEA) and staphylokinase (SAK), and a 20.2 kb phage remnant with homology to φNM4 of *S. aureus* strain Newman. Comparative genomic analysis indicates that isolate ED84 has lost the β-converting phage whereas isolate ED86 lacks the φSa2 homologue. These data indicate that phage deletion and/or acquisition events occur during *S. aureus* infection of the CF airways, consistent with previous findings [30]. By maintaining phages that encode secreted virulence factors in a subpopulation of the infecting bacteria, the metabolic costs to the infecting population as a whole are reduced in nutritionally-starved niches. Consistent with absence of the β-converting phage, strain ED84 demonstrated hemolytic activity for sheep erythrocytes, indicating the presence of a functional β-toxin gene (Fig. 1).

Overall, the genome-scale identification of polymorphisms affecting antibiotic resistance, growth, and global regulation of virulence indicate a profound impact on the ecology of *S. aureus* during chronic infection. The identification of loci under selective pressure during chronic *S. aureus* infection may indicate novel therapeutic targets for the control of persistent infections which are refractory to treatment.

## Supporting Information

Table S1 Genetic polymorphisms identified among the CF *S. aureus* isolates.
(DOC)
