# Supplementary material for: Adaptive Evolution of Staphylococcus aureus during Chronic Endobronchial Infection of a Cystic Fibrosis Patient
Source: PLoS One. 2011 Sep 2;6(9):e24301. doi: 10.1371/journal.pone.0024301 (PMC3166311; doi:10.1371/journal.pone.0024301)
Supplement: Table S1 — Genetic polymorphisms identified among the CF S. aureus isolates. (DOC) [file pone.0024301.s001.doc]

Table S1. Genetic polymorphisms identified among the CF *S. aureus* isolates

| Isolate | Polymorphism | Polymorphism effect^1^ | Locus |
| --- | --- | --- | --- |
| ED83 | Point mutation | NSS | Hypothetical protein |
| ED83 | Point mutation | NSS | yycG (Sensor kinase protein) |
| ED83 | Point mutation | NSS | Putative cobalmin synthesis protein |
| ED83 | 2 point mutations in single codon | NSS | Putative acetyl transferase |
| ED83 | Point mutation | NSS | sucA (2-oxoglutarate dehydrogenase E1 subunit) |
| ED83 | 1bp deletion | Frameshift affecting 46.6% of protein | Hypothetical membrane protein |
| ED83 | 1bp insertion | Frameshift affecting 18.5% of protein | rpsI (30S ribosomal protein S9) |
| ED83 | 1bp insertion | Frameshift affecting 80.8% of protein | Hypothetical protein |
| ED83, ED86 | 1bp deletion | - | Intergenic region |
| ED83 | 1bp deletion | Frameshift affecting 13.4% of protein | Hypothetical protein |
| ED83 | 1bp deletion | Frameshift affecting 23.1% of protein | phoR (alkaline phosphatase synthesis sensor protein) |
| ED83 | 1bp insertion | Frameshift affecting 36.7% of protein | sigB (RNA polymerase sigma factor SigB) |
| ED84 | Point mutation | - | Intergenic region |
| ED84 | Point mutation | NSS | ABC transporter ATP-binding protein |
| ED84 | Point mutation | NSS | glmM (putative phsphoglucosamine mutase) |
| ED84 | Point mutation | - | Intergenic region |
| ED84 | Point mutation | NSS | fnbA (Fibronectin-binding protein precursor A) |
| ED84 | Point mutation | - | Intergenic region |
| ED84 | 1bp deletion | Frameshift affecting 89.0% of protein | spoVG (regulatory protein SpoVG) |
| ED84 | 18bp deletion | Deletion of 6 amino acids | rsbU (putative sigma factor sigB regulation protein) |
| ED84 | 1bp insertion | Frameshift affecting 85.4% of protein | Hypothetical protein |
| ED84 | 1bp insertion | - | Intergenic region |
| ED84 | 1bp insertion | Frameshift affecting 46.3% of protein | Putative membrane protein |
| ED86 | Point mutation | NSS | spoVG (Regulatory protein SpoVG) |
| ED86 | Point mutation | NSS | fus (Elongation factor G) |
| ED86 | Point mutation | NSS | fus (Elongation factor G) |
| ED86 | Point mutation | SS | Hypothetical membrane protein |
| ED86 | Point mutation | NSS | codY (Transcriptional repressor CodY) |
| ED86 | Point mutation | NSS | infB (Translation initiation factor IF-2) |
| ED86 | Point mutation | NSS | Prephenate dehydrogenase |
| ED86 | Point mutation | NSS | Putative peptidase |
| ED86 | Point mutation | NSS | ald1 (Alanine dehydrogenase) |
| ED86 | Point mutation | NSS | moaB (Putative molybdenum cofactor biosynthesis protein B) |
| ED86 | Point mutation | NSS | Transcriptional regulator (antiterminator) |
| ED86 | 56bp deletion | - | Intergenic region |
| ED86 | 1bp deletion | Frameshift affecting 97.4% of protein | Hypothetical membrane protein |
| ED86 | 1bp insertion | Frameshift affecting 41.2% of protein | arlS (Sensor kinase protein) |

^1^NSS; Non-synonymous substitution, SS; Synonymous substitution
